# *Trypanosoma cruzi* P21: a potential novel target for chagasic cardiomyopathy therapy

**DOI:** 10.1038/srep16877

**Published:** 2015-11-17

**Authors:** Thaise Lara Teixeira, Fabrício Castro Machado, Aline Alves da Silva, Samuel Cota Teixeira, Bruna Cristina Borges, Marlus Alves dos Santos, Flávia Alves Martins, Paula Cristina Brígido, Adele Aud Rodrigues, Ana Flávia Oliveira Notário, Bruno Antônio Ferreira, João Paulo Silva Servato, Simone Ramos Deconte, Daiana Silva Lopes, Veridiana Melo Rodrigues Ávila, Fernanda de Assis Araújo, Tatiana Carla Tomiosso, Marcelo José Barbosa Silva, Claudio Vieira da Silva

**Affiliations:** 1 Instituto de Ciências Biomédicas, Universidade Federal de Uberlândia, MG, Brasil; 2 Departamento de Microbiologia, Imunologia e Parasitologia, Universidade Federal de São Paulo, SP, Brasil; 3 Instituto de Genética e Bioquímica, Universidade Federal de Uberlândia, MG, Brasil

## Abstract

Chagas disease, which is caused by the parasite *Trypanosoma cruzi,* is an important cause of cardiomyopathy in Latin America. It is estimated that 10%–30% of all infected individuals will acquire chronic chagasic cardiomyopathy (CCC). The etiology of CCC is multifactorial and involves parasite genotype, host genetic polymorphisms, immune response, signaling pathways and autoimmune progression. Herein we verified the impact of the recombinant form of P21 (rP21), a secreted *T. cruzi* protein involved in host cell invasion, on progression of inflammatory process in a polyester sponge-induced inflammation model. Results indicated that rP21 can recruit immune cells induce myeloperoxidase and IL-4 production and decrease blood vessels formation compared to controls *in vitro* and *in vivo*. In conclusion, *T. cruzi* P21 may be a potential target for the development of P21 antagonist compounds to treat chagasic cardiomyopathy.

Chagas disease, which is caused by the parasite *Trypanosoma cruzi,* is an important cause of cardiomyopathy in Latin America. It is estimated that 10%–30% of all infected individuals will acquire chronic chagasic cardiomyopathy (CCC). This represents anywhere between 1.6 to 5.4 million CCC patients in Latin America^1^. CCC has been shown to cause social and economic burdens in endemic areas because of increased health care costs^2^. An estimated 20.000 deaths occur annually in endemic countries due to complications of CCC^1^. The prognosis for chagasic patients is rather bleak. In fact, CCC has been reported to be the main prognostic mortality factor among patients with heart failure of various etiologies^1^,^2^.

The etiology of CCC is multifactorial and involves parasite genotype^3^,^4^, host genetic polymorphisms^5^,^6^,^7^,^8^,^9^, immune response^10^,^11^,^12^,^13^, signaling pathways^14^ and autoimmunity^15^,^16^. An intriguing question remains unanswered: could trypanosome-derived components play an active role in CCC onset rather than serving as passive targets for the host immune response ? In this sense, trapped intracellular parasites may continue to secrete proteins that can enter the extracellular space after plasma membrane wounding and/or lysis and influence disease progression. Herein we verified the impact of the recombinant form of P21 (rP21), a secreted *T. cruzi* protein involved in host cell invasion^17^,^18^,^19^, on progression of chronic inflammatory processes in a polyester sponge-induced inflammation model.

## Results and Discussion

### rP21 treatment increased IL-4 expression in polyester sponge-induced inflammation

**Figure 1:**
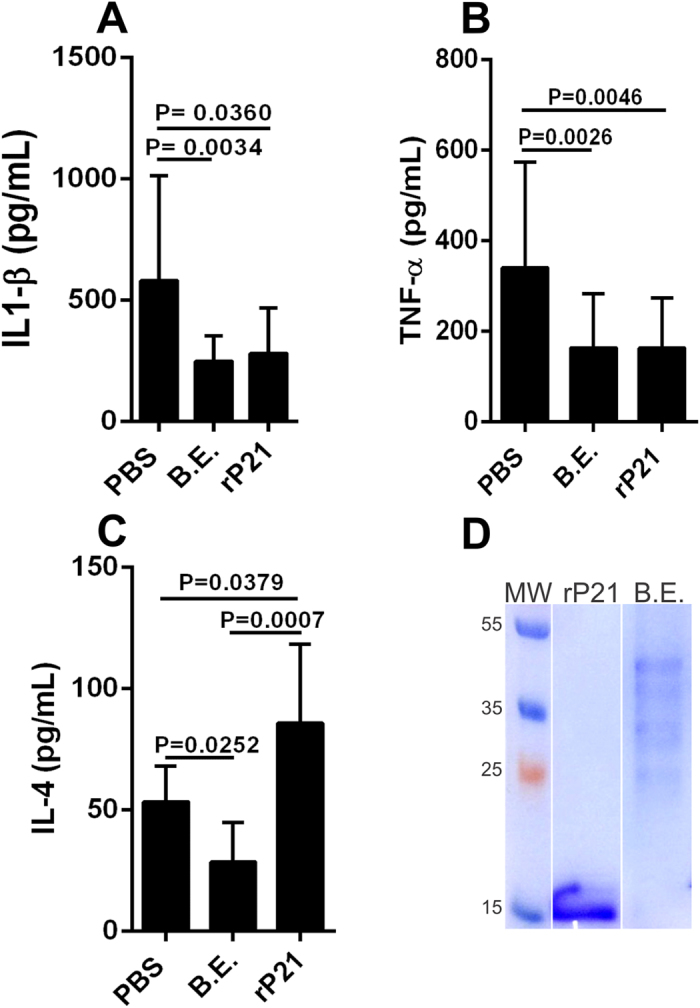
rP21 treatment increased IL-4 expression in polyester sponge-induced inflammation. rP21 and bacterial extract (B.E.) down-regulated interleukin-1β (IL-1β) **(A)** and tumor necrosis factor-α (TNF-α) **(B)**. B.E. inhibited IL-4 expression and rP21 treatment augmented this cytokine levels **(C)**. 40 μg/mL of rP21 and B.E. were used. The experiment was performed twice using 10 animals/group. Samples were analyzed individually in triplicate. Data are expressed as mean ± standard deviation. Significant differences were determined by Dunn’s test (GraphPad Prism software, version 6.01). Differences were considered significant when p < 0.05. A 12% Coomassie Blue stained Sodium dodecyl sulfate-Polyacrylamide gel electrophoresis (SDS-PAGE) gel is shown to demonstrate de purity of purified rP21 and the bacterial protein pattern in B.E. **(D)**. MW: molecular weight expressed in kilo Daltons (kDa). rP21 = 18 kDa.

We observed that both rP21 and bacterial extract (B.E.) down-regulated interleukin-1β (IL-1β) and tumor necrosis factor-α (TNF-α) expression in *in vivo* polyester sponge-induced inflammation (Fig. 1A,B). However, while B.E. inhibited IL-4, rP21 treatment augmented this cytokine levels (Fig. 1C). Knowledge of the pathology and immune response to *T. cruzi* infection has been largely obtained from murine models. These models have shown that the innate and adaptive immune responses play an important role in parasite control, depending on the combined action of various cellular types including NK, CD4+ and CD8+ as well as on the production of antibodies by B cells. Resistance to *T. cruzi* infection has been associated with the production of the pro-inflammatory cytokines IL-12 and IFN-*γ* and with the local production of RANTES, MIP-1*α*, MIP-1*β* and MCP-1. These cytokines activate the production of nitric oxide by macrophages, which is responsible for elimination of the parasite. TNF-*α* has also been associated with macrophage activation as a secondary signal for nitric oxide production. In contrast, cytokines such as IL-4 and TGF-*β* are associated with parasite susceptibility (For review^20^). *In vivo* and *in vitro* studies have established a clear role for IL-4 in driving T-helper 2 (Th2) immune response^21^.

Several studies^22^,^23^,^24^ demonstrated the importance of antibodies for survival and parasite clearance. Brener^25^ proposed that trypanolytic antibodies elicited by an active infection are the major and possibly the sole immune effector mechanism controlling murine and human *T. cruzi* infection. Although parasite-specific antibodies are essential for controlling *T. cruzi* infection, it has been described that B cells from acutely *T. cruzi*-infected mice showed a reduced reactivity to parasite antigens *in vitro*^26^. Minoprio *et al.*^27^ proposed that the humoral immunosuppression to parasite antigens observed during the acute phase of the infection might be due to the fact that parasite-specific B cells are outnumbered by polyclonally activated cells which produce non-specific antibodies that fail to bind parasite antigens. In addition, Hayes & Kierszenbaum^28^ described that B cells isolated only at the acute phase of the infection show an impaired reactivity to polyclonal activators despite the significant number of B lymphocytes, indicating a B cell alteration, a suppressive phenomenon, or a combination of both. In this context, it has been reported that stimulated B lymphocytes display reduced IL-2R expression in the presence of *T. cruzi* trypomastigotes^29^. Other researchers have suggested that a membrane antigen of *T. cruzi* is involved in immunosuppression^30^. Also, it has been demonstrated that polyclonal B cell activation during acute experimental infection is not a generalized response^31^. Altogether, these findings provide evidence for the coexistence of B cell immunosuppression and polyclonal activation during acute *T. cruzi* infection. The coexistence of these apparently contradictory immunological phenomena is consistent with the hypothesis that a continuous stimulation of lymphocytes leads them to an immune dysfunction: a loss of ability to respond to an antigen and/or an increase in the levels of apoptosis. In this sense, B cell apoptosis and cell cycle arrest could be the mechanisms that control intense B cell expansion, but at the same time could be delaying the emergence of a specific immune response against the parasite^32^. Moreover, authors have shown that IL-4 reduces B cells apoptosis in *T. cruzi* infected mice, blocks the differentiation of these cells to plasma cells, favoring the development of a memory B cell phenotype in a galectin-3-dependent pathway. This phenotype results in reduced immunoglobulin production and parasite clearance^33^. In this context, P21-driven IL-4 secretion may have impact on promoting a suitable environment for *T. cruzi* infection.

A 12% Coomassie Blue stained Sodium dodecyl sulfate-Polyacrylamide gel electrophoresis (SDS-PAGE) gel is shown to demonstrate de purity of purified rP21 and the bacterial protein pattern in B.E. (Fig. 1D).

### rP21 promoted myeloperoxidase expression and leukocytes chemotaxis

**Figure 2:**
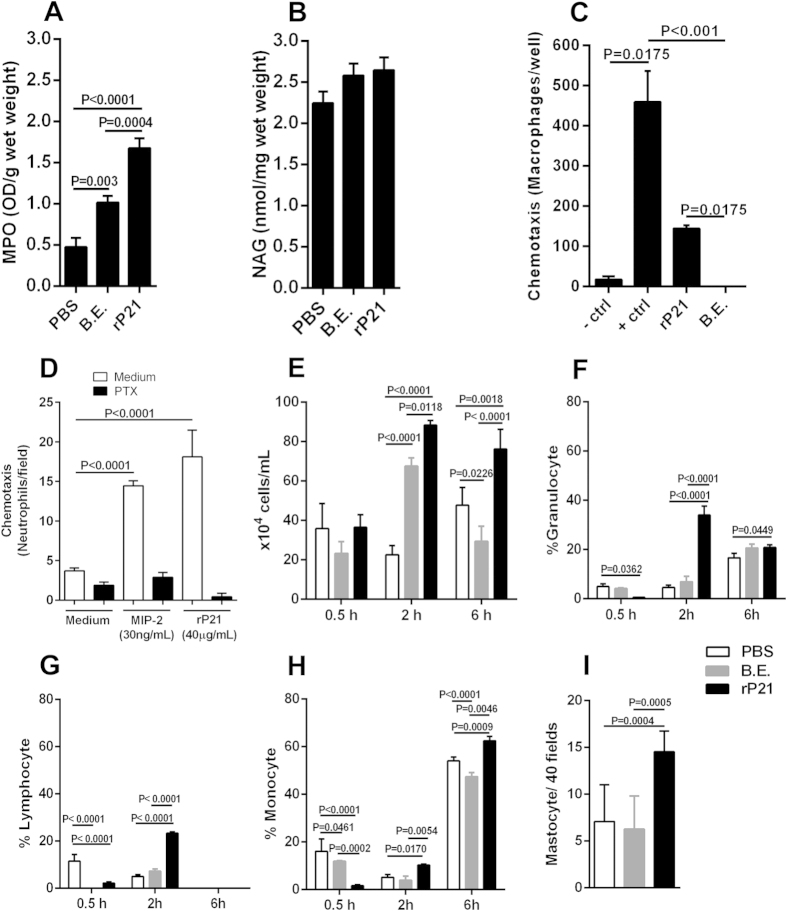
rP21 promoted myeloperoxidase expression and leukocytes chemotaxis. rP21 treatment enhanced the levels of myeloperoxidase (MPO) **(A)** but not of N-acetyl-beta-D-glucosaminidase (NAG) **(B)**. rP21 but not B.E. induced macrophage migration in a trans-well system. Negative control (-control): serum-free medium; Positive control (+control): stromal cell-derived factor 1 (SDF-1α – CXCL12) **(C)**. rP21 promoted neutrophil chemotaxis in Boyden chamber. This activity was completely abolished by previous cell treatment with pertussis toxin (PTX; negative control). Positive control: macrophage inflammatory protein (MIP-2) Experiments were performed four times in triplicate **(D)**. rP21 treatment induced the recruitment of higher number of total leukocytes to mice peritoneal cavity **(E)**. Phenotypic cell identification showed that at two hours rP21 promoted higher recruitment of granulocytes **(F)**, lymphocytes **(G)** and monocytes **(H)**. The ability of rP21 to recruit higher number of monocytes extended up to six hours post-inoculation. Higher number of mastocytes was observed in rP21-treated sponges compared to PBS and B.E. treatments. Experiments were performed twice using 6 animals/group. Samples were analyzed individually **(I)**. 40 μg/mL of rP21 and B.E. were used. Data are expressed as mean ± standard deviation of experiments performed in triplicate. Significant differences were determined by one-way **(A,B,C,D,I)** or two-way ANOVA **(E,F,G,H)** and Turkey’s multiple comparisons test (GraphPad Prism software, version 6.01). Differences were considered significant when p < 0.05.

rP21 treatment enhanced the levels of myeloperoxidase (MPO) but not of N-acetyl-beta-D-glucosaminidase (NAG) compared to control treatments in polyester sponge-induced inflammation (Fig. 2A,B). Moreover, the inflammatory score analysis showed that polyester sponges treated with rP21 displayed moderate inflammatory infiltrate, mostly composed of macrophages. B.E. treated sponges exhibited intense inflammatory infiltrated mainly composed of neutrophil and lymphocytes with a moderate level of necrotic tissue (Table 1). MPO is a lysosomal enzyme produced in high amounts by neutrophils, especially during their early maturation phase. MPO catalyzes the production of hypochlorous acid from hydrogen peroxide and chloride anion and oxidizes tyrosine to tyrosyl radicals. These radicals are cytotoxic to a variety of microorganisms. However, MPO released into extracellular milieu also contributes to tissue injury and disease pathology^34^. The lack of correlation between MPO levels and neutrophil infiltrate in rP21 treated sponges may be in part due to the expression of this enzyme by macrophages and B-lymphocytes^35^. Moreover, authors have identified granulocyte macrophage colony-stimulating factor as an endogenous regulator of macrophage myeloperoxidase expression in human atherosclerosis^36^.

Our previous studies showed that rP21 enhanced macrophage phagocytosis and actin polymerization by binding to the CXCR4 receptor^18^. The ability of rP21 to bind to CXCR4 indicated a potential chemotaxis activity driven by this protein. We performed different *in vitro* and *in vivo* experiments in order to gain insight into the direct effect of rP21 over leukocyte migration. First, we addressed the ability of rP21 to induce migration of peritoneal macrophages in a trans-well system through a polycarbonate membrane over a thin layer of extracellular matrix. Our results showed that rP21 but not B.E. induced macrophage migration (Fig. 2C). After, we verified that rP21 promoted neutrophil chemotaxis in Boyden chamber. This activity was completely abolished by previous cell treatment with pertussis toxin (PTX) (Fig. 2D). Finally, to assess whether rP21 has chemo-attractive activity *in vivo*, it was injected intra-peritoneally into mice and we observed cell recruitment at different time point compared to injection of PBS or B.E. The results showed that at two and six hours post-inoculation, rP21 treatment induced the recruitment of higher number of total leukocytes compared to the control treatments (Fig. 2E). Phenotypic cell identification showed that at two hours rP21 promoted higher recruitment of granulocytes, lymphocytes and monocytes (Fig. 2F,G). Leukocytes recovered from peritoneal cavity previous to PBS, B.E. or rP21 inoculation was similar to the result obtained at 0.5 hour post-inoculation (data not shown). The ability of rP21 to recruit higher number of monocytes extended up to six hours post-inoculation (Fig. 2H).

The literature has shown that the inflammatory process that characterizes CCC is accentuated during the acute phase of the disease. Although it may be clinically silent after the acute phase, inflammation is continuously present in patients with indeterminate and chronic forms of the disease^37^,^38^. Moreover, trypanosome antigens and/or its genomic material are found in inflammatory foci^39^,^40^,^41^,^42^,^43^. These observations provide a basis to propose a role for P21 regarding the onset and maintenance of chagasic heart inflammation.

Also, we observed higher number of mastocytes in rP21-treated sponges compared to PBS and B.E. treatments (Fig. 2I). This result may be in part explained by the higher levels of IL-4 detected in rP21-treated sponges. IL-4 is known to enhance mastocytes maturation, survival, proliferation and migration^44^. Mastocytes have been implicated in cardiovascular dysfunctions, such as ischemic heart disease, experimental myocardial infarction, myocarditis, heart failure, transplant-related fibrosis, and hypertensive heart disease^45^,^46^,^47^,^48^. *In vitro* and *in vivo* studies have shown the presence of mastocytes associated with cardiac *T. cruzi* infection^49^,^50^,^51^.

### rP21 inhibited angiogenesis *in vivo* and *in vitro*

**Figure 3:**
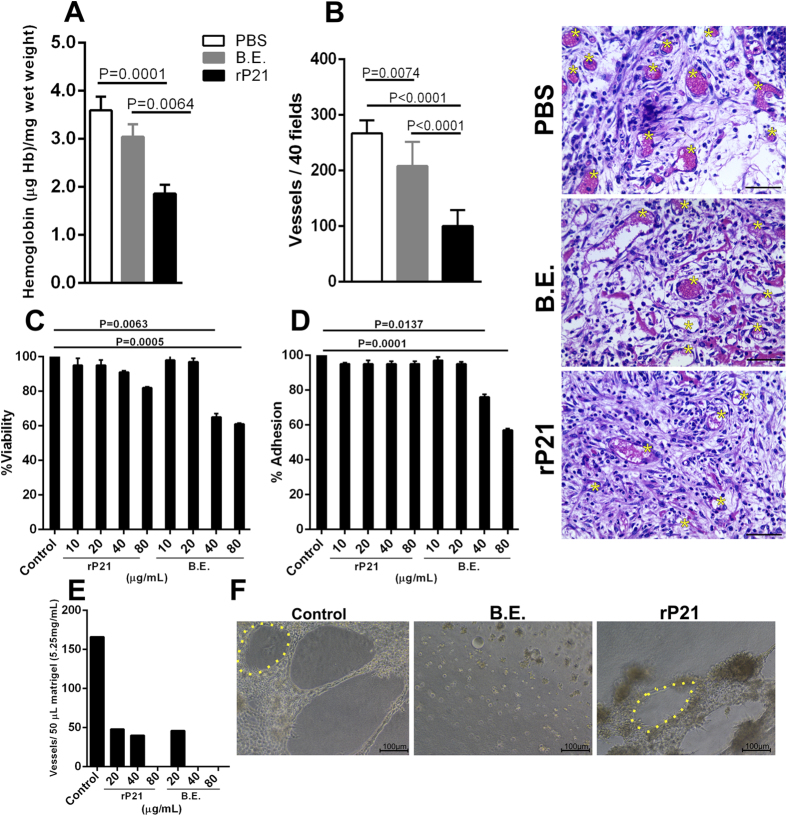
rP21 inhibited angiogenesis *in vivo* and *in vitro*. Decrease in hemoglobin content was observed in rP21 treated sponges **(A)**. Sponges treated with rP21 showed a decreased number of blood vessels compared to the control treatments **(B)**. Representative HE-stained sponges are shown (panel on the up-right position) and yellow asterisks indicate blood vessels. 40 μg/mL of rP21 and B.E. were used. rP21 did not alter tEnd cell viability **(C)**, adhesion **(D)** to extracellular matrix and inhibited vessel formation **(E)**. B.E. showed cell toxicity and inhibition of adhesion at the concentrations of 40 and 80 μg/mL (representative results of four independent experiments performed in triplicate). Representative images highlighting vessels morphology (yellow dotted line) are also shown **(F)**. Data are expressed as mean ± standard deviation of experiments performed in triplicate. Significant differences were determined by one-way ANOVA and Turkey’s multiple comparisons test (GraphPad Prism software, version 6.01). Differences were considered significant when p < 0.05.

We observed a significant decrease in hemoglobin content in rP21 treated sponges (Fig. 3A). This result suggested a potential anti-angiogenic role of rP21. To confirm this hypothesis, we determined the number of blood vessels on histological preparations. Sponges treated with rP21 showed a decreased number of blood vessels compared to the control treatments (Fig. 3B). We also treated a murine endothelial cell line derived from thymus hemangioma (tEnd) with different concentrations of rP21 or B.E. and observed that rP21 did not alter cell viability and adhesion to a thin layer of extracellular matrix (Fig. 3C,D) but strongly inhibited vessel formation (Fig. 3E). B.E. showed cell toxicity and inhibition of adhesion at the concentrations of 40 and 80 μg/mL (Fig. 3C–E). The toxicity and inhibition of cell adhesion observed in B.E. treated cells may explain the lack of vessel formation. Representative images highlighting cell and vessels morphologies are also shown (Fig. 3F). The anti-angiogenic activity of rP21 may be due to a cascade of events triggered by direct interaction of the protein to endothelial cells. This possibility will be addressed in novel studies from our research group. However, another non excluding hypothesis is that rP21-induced IL-4 drives macrophage to produce soluble fms-like tyrosine kinase 1 (sFlt-1) and express an Arginase-1(+) phenotype resulting in the inhibition of angiogenesis^52^.

Functional and structural micro-vascular abnormalities occur in CCC^53^,^54^. Consequently, vasospasms, decreased blood flow, focal ischemia, platelet thrombi, increased platelet aggregation, and elevated levels of thromboxane A-2 and endothelin-1 are frequently observed^55^,^56^,^57^,^58^. As such, P21 could impact on micro-vascular ischemia, which is believed to amplify chronic inflammatory aggression toward myocardial tissue^59^.

### Envisaged mechanism triggered by rP21 in inflammation

**Figure 4:**
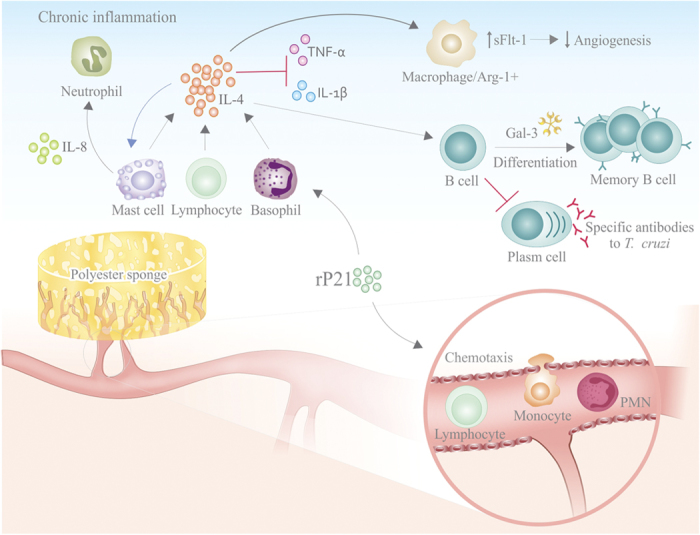
Envisaged mechanism triggered by rP21 in inflammation. rP21 induces recruitment of leukocytes to the site of inflammation and up-regulates the expression of IL-4. IL-4 is produced by lymphocytes, basophils and mastocytes and promotes a Th2 immune response. Also, IL-4 induces macrophages to acquire an Arginase-1(+) phenotype and to increase production of sFlt-1 that culminates in the inhibition of angiogenesis. Moreover, IL-4 promotes survival and differentiation of B-lymphocytes in memory cells negatively impacting on immunoglobulin production and secretion. Mastocytes in the inflammatory foci will liberate several bioactive molecules that may participate in tissue damage, recruitment of neutrophils and amplification of IL-4-driven pathways.

Taken our results together with the current literature we propose that rP21 induces recruitment of leukocytes to the site of inflammation and up-regulates the expression of IL-4. IL-4 is produced by lymphocytes, basophils and mastocytes and promotes a Th2 immune response. Also, IL-4 induces macrophages to acquire an Arginase-1(+) phenotype and to increase production of sFlt-1 that culminates in the inhibition of angiogenesis. Moreover, IL-4 promotes survival and differentiation of B-lymphocytes in memory cells negatively impacting on immunoglobulin production and secretion. Mastocytes in the inflammatory foci will liberate several bioactive molecules that may participate in tissue damage, recruitment of neutrophils and amplification of IL-4-driven pathways (Fig. 4).

This putative mechanism suggests a potential role of native P21 during *T. cruzi* pathogenesis and highlights P21 as a novel target for the development of innovative therapeutics agents against CCC.

## Experimental procedures

### Animals and ethics

Six-to-eight-week-old male C57BL/6 mice were maintained under standard conditions on a 12-h light-dark cycle in a temperature controlled setting (25 °C) with food and water *ad libitum*. Maintenance and care of animals complied with the guidelines of the Laboratory Animal Ethics Committee from the Universidade Federal de Uberlândia. Animal euthanasia was performed based on international welfare grounds according to the American Veterinary Medical Association Guidelines on Euthanasia. This study was approved by the ethics committee for animal research at Universidade Federal de Uberlândia.

### Cell culture

Murine endothelial cell lines derived from thymus hemangioma (tEnd)^60^ were cultivated in RPMI 1640 medium supplemented with 10% bovine fetal serum, 2 mM l-glutamine, 2 mM sodium pyruvate, 1 mM non-essential amino acids, 100 U/mL penicillin and 100 μg/mL streptomycin and incubated at 37 °C and 5% CO_2_.

### rP21 purification

rP21 (GenBank: EU004210.1) was purified as previously described^17^,^19^. B.E was obtained under the same conditions without inducing rP21 expression. The quality of purification was demonstrated by SDS-PAGE. Similar concentration of rP21 and B.E. was used in the experimental procedures.

### Polyester inflammation induction and treatment scheme

A polyester sponge with 1.2 cm diameter (Vitafoam Ltd.) was stored in 70% ethanol v/v for at least 24 hours and subsequently boiled in distilled water for 30 minutes^61^,^62^. C57BL/6 mice were divided into groups of 10 animals for biochemical and 6 animals for histological analysis, treated with PBS, 40 μg/mL of rP21^17^,^18^,^19^ or B.E. The animals were anesthetized via intra-peritoneal with ketamine-xylazine (Syntec) (60 mg–4 mg/kg) and subjected to trichotomy and antisepsis in the dorsal region with alcohol 70% v/v. The sponge was introduced by midline incision in the inter-scapular region. Incisions were closed with a Donati suture using nylon 3.0. After recovery from anesthesia, animals were placed in individual cages with food and water *ad libitum*^50^. Treatments were injected into the sponge every 72 hours and analyzed during the chronic (9 days) phase of inflammation.

### Hemoglobin content

Determination of the intra-implant hemoglobin content was performed using Drabkin’s reagent^61^,^62^. Implants were removed and mass determined 9 days post-implantation. Samples that showed macroscopic bleeding or infection were excluded from analysis. Each implant was homogenized (Tekmar TR-10, Ohio, USA) in 2 mL of a hemoglobin-specific chromogen reagent (Drabkin kit, Labtest) and added to 2 mL tubes. Samples were centrifuged at 4 °C for 30 minutes at 12,000 × g and homogenates were filtered through 0.22 μm filters (Millipore). Spectrophotometric analysis was performed at 540 nm using 96-well plates. The hemoglobin concentration of each sample was calculated based on a standard curve and the results were expressed as hemoglobin concentration (micrograms) per milligram wet weight of the implant.

### Myeloperoxidase (MPO) activity

After determining the hemoglobin content, the supernatant was stored in protease inhibitor cocktail (Sigma Aldrich) at −80 °C. The pellet was resuspended in 2 mL sodium phosphate buffer (pH 5.4). 300 μL was transferred to tubes and supplemented with 600 μL of 0.5% w/v HTAB (hexadecyltrimethylammonium bromide, Sigma) diluted in phosphate buffer (pH 5.4). After, samples were centrifuged at 10,000 × g for 10 minutes at 4 °C and the supernatant was used. Finally, 100 μL of 0.003% hydrogen peroxide and 100 μL TMB (3,3′,5,5′-tetramethylbenzidine, Sigma) at 4 mM diluted in DMSO (dimethyl sulfoxide, Merck) were added in a novel tube followed by 200 μL of the sample supernatant and incubated for 1 minute. The reaction was stopped by adding 100 μL of 4 M H_2_SO_4_ (sulfuric acid, Merck). 200 μL of the final solution was added to 96-well plate and spectrophotometric analysis was performed at 450 nm. Results are expressed as the MPO index (absorbance/mg wet weight of the implant).

### N-acetyl-beta-D-glucosaminidase (NAG) activity

After determining the hemoglobin content, the supernatant was stored in protease inhibitor cocktail (Sigma Aldrich) at −80 °C. The pellet was resuspended in 2.0 mL 0.9% saline with 0.1% Triton X-100 (Promega) and centrifuged at 3000 × g for 10 minutes at 4 °C. 100 μL of the supernatant was added to a 96-well plate in duplicate. After, 100 μL of substrate (p-nitrophenyl-N-acetyl-beta-D-glucosaminide, Sigma) diluted in citrate /phosphate buffer pH 4.5 was added to the samples and incubated at 37 °C for 30 minutes. Finally, 100 μL of 0.2 M glycine buffer pH 10.6 was added and absorbance was measured in spectrophotometer at 400 nm. NAG activity was calculated using a standard curve of p-nitrophenol. Results are expressed as nmol mL-1/mg wet weight of the implant.

### Cytokines detection

The supernatant obtained after determining the hemoglobin content was used to measure the levels of IL-1β, TNF-α and IL-4 by Enzyme-Linked Immuno Sorbent Assay (ELISA) according to the manufacturer (BD Biosciences).

### Histological analysis

Sponges designated for histological analysis were fixed in Metacarn buffer (60% methanol, 30% chloroform, 10% acetic acid) for 3 hours at 4 °C. Sponges were prepared for inclusion in ethyl alcohol PA for 30 minutes with 4 washes in xylene, followed by additional 30 minutes of incubation, three washes and finally embedded in paraffin. The blocks were processed for hematoxylin and eosin (HE) and toluidine blue staining. Images were obtained in a Leica DM500 microscope coupled to Las Ez camera and software. The type and severity of the inflammatory infiltrate were determined by the presence or absence of (i) neutrophils, (ii) macrophages, (iii) lymphocytes, (iv) plasmocytes, (v) necrotic tissue, (vi) intensity of inflammatory response. These features were scored for intensity: (−) absent, (+) mild, (++) moderate, (+++) intense.

### Cell viability and adhesion

The viability of tEnd cells treated with rP21 was evaluated using the MTT (3-(4,5-dimethylthiazol-2-yl)-2,5-diphenyl tetrazolium bromide) assay. Cells were seeded at 1.5 × 10^4^ cells/well in 96-well micro-plates. After adhesion, cells were treated with different concentrations of rP21, Bacterial Extract (B.E.) or culture medium for 24 hours at 37 °C and 5% CO_2_. After 24 hours, cells were incubated with 5 mg/mL MTT for 3 hours at 37 °C. Formosan crystals resulting from MTT reduction were dissolved by addition of 100 μL of PBS containing 10% SDS and 0.01 M HCl (18 h, 37 °C and 5% CO_2_). Absorbance (570 nm) was measured on a multi-well scanning spectrophotometer (Thermo Scientific).

For adhesion assay, tEnd cells (2 × 10^4^ cells/well) were pre-incubated for 30 min at 37 °C with different concentration of rP21, B.E. or culture medium. After, cells were added to 96-well plate at 37 °C and 5% CO_2_. After 2 h, detached cells were removed by two washes using PBS. Finally, the plate was incubated with 5 mg/mL MTT for 3 h at 37 °C, as described above.

### Angiogenesis assay

The influence of rP21 on endothelial cell tube formation was evaluated by Matrigel tube formation assays. Experiments were performed as described^63^. tEnds cells (2 × 10^5^ cells/well) were pre-incubated with 10, 40 and 80 μg/mL of rP21, B.E. or only culture medium for 30 minutes at room temperature and plated on 24-well plates previously coated with 250 μL of 5.25 mg/mL Matrigel (BD Bioscience) and supplemented with basic Fibroblast Growth Factor (bFGF) (30 ng/mL). After 18 hours of incubation at 37 °C and 5% CO_2_, images were acquired at×20 magnifications in bright light microscope.

### *In vitro* and *in vivo* chemotaxis assays

The *in vitro* chemotaxis assay for peritoneal macrophages was performed in a trans-well system through a polycarbonate membrane with a defined pore size of 8 μm (Chemicon Cell Invasion Assay Kit, EMD Millipore Corporation, Darmstadt, Alemanha). Peritoneal macrophages from C57BL/6 mice were seeded at 10^6^ in top of the insert in serum-free medium. Serum-free medium contained 40 μg/mL of rP21 or B.E. or stromal cell-derived factor 1 (SDF-1α–CXCL12–50 ng/mL–Sigma Aldrich) or medium only was added in the bottom well. Cells were incubated for 72 hours. Migratory cells were stained and counted using an optical microscope.

For neutrophil chemotaxis, a Boyden chamber assay was used, consisting of upper and lower chambers separated by a polycarbonate membrane with an 8-μm pore. Neutrophils were extracted from C57BL/6 bone marrow using Ficoll-Paque (Sigma Aldrich) to separate them from other cells. Some of the cells were pre-treated with pertussis toxin (PTX – 100 ng/mL–Sigma Aldrich) (G-protein coupled receptor inhibitor) for 1 hour. Treated and non-treated cells were placed in the upper chamber. In the lower chamber, only media, rP21 or macrophage inflammatory protein (MIP-2–30 ng/mL–Life Technologies), a chemotaxis positive control, were added. After 1 hour, the membrane was removed, fixed and colored with panoptic dye. Cells that migrated were counted using an optical microscope.

*In vivo*, C57BL/6 mice received three different intra-peritoneal treatments: PBS, 40 μg/mL of rP21^17^,^18^,^19^ or B.E. The animals were euthanized at intervals of 0.5, 02 and 06 hours. Peritoneal lavage was obtained by inoculating 4 mL PBS into the peritoneum cavity. Total leukocyte recruitment was quantified in a Neubauer chamber. Cells were phenotypically identified by size and granularity in flow cytometry (BD FacsCanto II).

### Statistical analysis

Data are expressed as mean ± standard deviation of experiments performed in triplicate. Significant differences were determined by one-way or two-way ANOVA and Turkey’s multiple comparisons test (parametric) or Dunn’s test (nonparametric), according to the experimental design (GraphPad Prism software, version 6.01). Differences were considered significant when p < 0.05.

## Additional Information

**How to cite this article**: Teixeira, T. L. *et al.* *Trypanosoma cruzi* P21: a potential novel target for chagasic cardiomyopathy therapy. *Sci. Rep.* **5**, 16877; doi: 10.1038/srep16877 (2015).

## Figures and Tables

**Table 1:** Qualitative analyses of cell infiltrate in sponges treated with PBS, B.E or rP21.

Histological criteria	PBS	B.E.	rP21
Neutrophils	+	+++	+
Macrophages	++	++	+++
Lymphocytes	+++	+++	++
Plasma cells	++	+	+
Necrotic tissue	−	++	+
Inflammatory response	++	+++	++
